# Applications of single-cell sequencing in cancer research: progress and perspectives

**DOI:** 10.1186/s13045-021-01105-2

**Published:** 2021-06-09

**Authors:** Yalan Lei, Rong Tang, Jin Xu, Wei Wang, Bo Zhang, Jiang Liu, Xianjun Yu, Si Shi

**Affiliations:** 1 Department of Pancreatic Surgery, Fudan University Shanghai Cancer Center, No. 270 Dong’An Road, Shanghai, 200032 China; 2 Department of Oncology, Shanghai Medical College, Fudan University, Shanghai, China; 3 Shanghai Pancreatic Cancer Institute, No. 270 Dong’An Road, Shanghai, 200032 China; 4 Pancreatic Cancer Institute, Fudan University, Shanghai, China

**Keywords:** Single-cell sequencing, Heterogeneity, Microenvironment, Circulating tumor cells

## Abstract

Single-cell sequencing, including genomics, transcriptomics, epigenomics, proteomics and metabolomics sequencing, is a powerful tool to decipher the cellular and molecular landscape at a single-cell resolution, unlike bulk sequencing, which provides averaged data. The use of single-cell sequencing in cancer research has revolutionized our understanding of the biological characteristics and dynamics within cancer lesions. In this review, we summarize emerging single-cell sequencing technologies and recent cancer research progress obtained by single-cell sequencing, including information related to the landscapes of malignant cells and immune cells, tumor heterogeneity, circulating tumor cells and the underlying mechanisms of tumor biological behaviors. Overall, the prospects of single-cell sequencing in facilitating diagnosis, targeted therapy and prognostic prediction among a spectrum of tumors are bright. In the near future, advances in single-cell sequencing will undoubtedly improve our understanding of the biological characteristics of tumors and highlight potential precise therapeutic targets for patients.

## Background

Single-cell sequencing, based on next-generation sequencing, has progressed rapid in recent years. The first single-cell mRNA sequencing experiment was conducted in 2009, the first single-cell DNA sequencing experiment in human cancer cells was conducted in 2011, and the first single-cell exome sequencing experiment was conducted in 2012 [1, 2]. Single-cell sequencing significantly outperforms previous sequencing technologies in terms of our understanding of the human biology of embryonic cells, intracranial neurons, malignant tumor cells and immune cells because it can probe cellular and microenvironmental heterogeneity at single-cell resolution. It has revolutionized our ability to interrogate the transcriptional, genomic, epigenomic and metabolic characteristics of thousands of individual cells in depth, thereby enabling an unbiased analysis of the cells within tumor lesions. It also provides molecular insights, including single-nucleotide variations (SNVs), copy number variations (CNVs) and structural variations (SVs) [3, 4]. However, the limitations of single-cell sequencing should not be neglected; these include its limited sensitivity, scale and accuracy; insufficiency in reconstructing clonal evolution in spatial and temporal order; and noise due to the preamplification of single-cell RNA. These limitations have mainly been overcome by improving the technology itself and combining it with other emerging technologies, enabling researchers to analyze multiomic information at a single-cell resolution.

Single-cell sequencing offers the ultimate resolution, thereby contributing to exploring effective management and personalized therapeutics in a tremendous number of scenarios, such as developmental research, the preparation of human cell atlases and cancer research [5, 6]. In addition to developmental biology, single-cell RNA sequencing (scRNA-seq) and its derived technologies might assist in the identification of novel markers, rare subgroups and evolution patterns, especially in brain development [7, 8]. In cancer research, it can be used to identify or interrogate (1) rare subpopulations; (2) circulating tumor cells (CTCs); (3) the tumor or immune microenvironment; (4) tumor heterogeneity and molecular subtype; (5) mechanisms associated with tumorigenesis, progression, metastasis, evolution, relapse and therapy resistance; and (6) cancer stem cells (CSCs) [9–12].

In this review, we summarize recent progress in single-cell sequencing technologies, including emerging single-cell sequencing technologies, single-cell sequencing data analysis, and applications in cancer research.

## Emerging single-cell sequencing technologies

Breakthroughs in single-cell isolation, sequencing, cDNA library preparation and analytical algorithms have led to rapid progress in single-cell sequencing analysis capacity. Single-cell transcriptomics, genomics, proteomics, epigenomics and interactomics sequencing have developed rapidly in recent years.

scRNA-seq technologies share common procedures, including (1) the isolation of a single cell, (2) RNA extraction, (3) reverse transcription, (4) preamplification and (5) detection [13]. The earliest procedures are clearly the most crucial, as they determine the accuracy and amount of the amplified material. A study showed that a lower single-cell dissociation temperature (6 °C) minimizes the stress responses induced at 37 °C, which includes the induction of 512 heat shock proteins. Moreover, the method of dissociation also affects transcription signatures [14].

In addition to the need for single-cell isolation, the greatest challenge in single-cell sequencing is recognizing the sequencing results at the single-cell level. Single-cell barcoding technologies based on plate microreaction systems and combinational indices have completely resolved this bottleneck, thus increasing the throughput of single-cell analysis by at least 100-fold. First, plate microreaction systems usually include single cells, functional beads and reverse transcriptomes. The surface of the functional beads is modified with oligonucleotides, including primers, cell barcodes, unique molecular identifiers (UMIs) and poly(dT) moieties from 5′ to 3′. The primer and poly(dT) moieties are uniform among the microreaction systems, while the cell barcode is unique for each microreaction system, and the UMI is unique for each molecule within a single cell. In addition, the UMI can label various molecules, such as the DNA genome, transcriptome, immune profile and proteome, based on different sequencing purposes. These features guarantee the precise identification of molecular features of a single cell. Drop-seq, Seq-Well and inDrop are bead-based technologies. Zhang et al. revealed the differences between Drop-seq and inDrop in detail. For example, the length of the cell barcode is 38–41 bp in inDrop and 12 bp in Drop-seq, and the cell barcode capacity is 147,456 (384*384) in inDrop and 16,777,216 (4^12^) in Drop-seq [15]. Additionally, the same type of bead is used in Seq-Well and Drop-seq [16]. Another single-cell barcoding technology based on combinational indexing overcomes the limitations of the relatively high cost of isolating a single cell for bead-based barcoding technologies. Combinational indexing-based barcoding technologies recognize single cells by the addition of cellular barcodes in multiple rounds without isolating single cells; these technologies include Sci-Seq, Microwell-Seq and Split-Seq [17]. In Sci-Seq, two rounds of transposase barcoding and PCR ligation labeled 9216 (96*96) single cells. Three split-pool rounds introduced 3 parts of the oligonucleotide sequence to the magnetic beads in Microwell-Seq [18]. However, too few cells for massive analysis are isolated in Sci-Seq and Microwell-Seq. Split-seq, which is based on Sci-Seq, utilizes 5 rounds of barcoding, which significantly improves sequencing over millions of single cells and lowers the cost [8].

After barcode-targeted molecules, the next important procedure is preamplification, in which the transcripts undergo reverse transcription. The preamplification procedure in 10X Genomics technology occurs as follows. First, the transcripts are bound to the 5′ poly(dT) in oligonucleotides by the polyA tail at its 3′ end. Second, the oligonucleotides undergo reverse transcription from the 5′ end to the 3′ end, with the binding transcript used as a template. The oligonucleotide chain extension ends with several C bases whose addition is catalyzed by a special terminal transferase. Third, the template switch oligo (TSO) is added to the template by reverse transcriptase, followed by the addition of the TSO to the other chain. Then, the new full-length cDNA, which is also known as the first-strand cDNA, becomes the new template for cDNA amplification, called the template switch. Fourth, the second cDNA chain is synthesized with the first cDNA chain used as a template [19]. The whole process is called in vitro transcription. In addition, multiple displacement amplification (MDA) is a non-PCR-type DNA amplification method that relies on isothermal amplification. MDA utilizes a special DNA polymerase named bacteriophage phi29 DNA polymerase, which can amplify very small amounts of DNA even from a single cell with a high binding capacity [20]. In addition, newly developed in situ 10-cell RNA sequencing, which utilizes Taq and Phusion polymerases, has dramatically increased the yield of ~ 500 bp preamplification products and enables a tenfold decrease in the T24-containing primer concentration without a detectable loss in preamplification efficiency. In addition, in situ 10-cell RNA sequencing allows the simultaneous sequencing of 10 microdissected cells in their normal tissue context [21].

For detection, most single-cell sequencing techniques rely on empirical parameters or predefined structures to estimate the degree of complexity. Recent research revealed that the combination of nonnegative matrix factorization and Bayesian model comparison with current algorithms enables unambiguous assessments of the depth of heterogeneity in the tumor microenvironment (TME) [22]. Single-cell hierarchical Poisson factorization can be used to discover both continuous and discrete expression patterns from de novo scRNA-seq data [23]; moreover, newly developed super CT can train the expandable supervised classifier once the RNA data are input [24]. The accurate interrogation of cell subsets contributes to a better understanding of the clonal composition and heterogeneity.

Moreover, we have summarized the most important single-cell sequencing technologies and platforms in Table 1 [25].

**Table 1:** Summary of important single-cell sequencing technologies and platforms

Measurement	Technology	Platform	Year	Single cell isolation	Gene coverage	Barcode addition	Library amplification	Reaction volume	Throughput	References
Transcriptome	SPLit-seq	Plated-based, Illumina NextSeq	2018	Not needed	3′	Ligation of barcoded RT primers	PCR	Microliter	High	[8]
	SCI-RNA-seq	Plate-based, Illumina NextSeq 500	2017	Not needed	3′	Barcoded RT primers	PCR	Not needed	High	[17]
	Seq-well	10X Genomics, Illumina NextSeq 500	2017	Droplet	3′	Barcoded RT primers	PCR	Nanoliter	High	[191]
	inDrop	Not mentioned	2015	Droplet	3′	Barcoded RT primers	In vitro transcription	Microliter	High	[192]
	Drop-seq	10X Genomics, Illumina NextSeq 500	2015	Droplet	3′	Barcoded RT primers	PCR	Nanoliter	High	[193]
	Microwell-Seq	Plate-based, Illumina HiSeq	2018	FACS	3′	Barcoded RT primers	PCR	Microliter	High	[18]
	MARS-seq	Plate-based	2014	FACS	3′	Barcoded RT primers	In vitro transcription	Microliter	High	[194]
	CEL-seq 2	Fluidigm C1, illumine TrueSeq	2016	FACS	3′	Barcoded RT primers	In vitro transcription	Microliter	Low	[195]
	SMART-Seq 2	Plate-based, Illumina HiSeq 2000	2013	FACS	Full length	Library PCR with barcoded primers	PCR	Microliter	Low	[196]
Genomics	TARGET-seq	Plate-based, Illumina NextSeq 500/550	2019	FACS	3′ and full-length	Barcoded RT primers	PCR	Microliter	High	[53]
Epigenomics	CoBATCH	10X Genomics; Illumina HiSeq X	2019	FACS	Full length	Barcoded PAT transposase	PCR	Microliter	High	[197]
	scATAC-seq	10X Genomics, Illumina NextSeq 500	2013	FACS	full length	Barcoded primers	PCR	Microliter	High	[198]
Transcriptomics, proteomics	CITE-seq	10X Genomics, Illumina HiSeq 2500	2017	Droplet	3′	Barcoded RT primers	PCR	Nanoliter	High	[199]
	REAP-seq	10X Genomics, Illumina HiSeq 2500	2017	FACS	3′	Barcoded RT primers, DNA-barcoded antibodies	PCR	Microliter	High	[200]
	INs-seq	10X Genomics, Illumina NextSeq 500	2020	FACS	5′	Barcoded RT primers	In vitro transcription	Microliter	High	[66]

RT: reverse transcription; SPLiT-seq: split-pool ligation-based transcriptome sequencing; SCI-Seq: single-cell combinatorial indexed sequencing; FACS: fluorescence-activated cell sorting; MARS-seq: massively parallel single-cell RNA sequencing; inDrop: indexing drop RNA sequencing; ScATAC-seq: single-cell transposase-accessible chromatin using sequencing; CoBATCH: combinatorial barcoding and targeted chromatin release; PAT: Tn5 transposase to protein A; CITE-seq: cellular indexing of transcriptomes and epitopes by sequencing; REAP-seq: RNA expression and protein sequencing assay; INs-seq: intracellular staining and sequencing

### Transcriptomics

Currently, scRNA-seq is widely applied to profile the transcriptomes of individual cells. Droplet-based 10X Genomics Chromium and plate-based Switching Mechanism at the 5′ End of RNA Template sequencing (SMART-Seq) are two frequently used platforms.

The 10X Genomics platform, which is based on a microfluidics approach, is capable of isolating, labeling, amplifying and preparing a cDNA library from 5000 to 10,000 single cells at a high speed. However, it detects only the 3′ or 5′ end of the transcript with a bias, and abundant cells in a single sample (recommended over 90%) are needed. Moreover, the cell capture rate is lower than that of SMART-Seq, so it is not suitable for detecting rare samples containing few cells.

SMART-Seq, which was reported in 2012, facilitates the detection of full-length transcripts [26]. SMART-Seq v2, which was reported in 2013, eliminates purification procedures, replaces the last guanosine at the TSO 3′ end with locked nucleic acid (LNA) and utilizes betaine to increase protein thermal stability, thus significantly increasing the yield [27]. Interestingly, the fundamental basis for full-length sequencing is Moloney murine leukemia virus (MLV) reverse transcriptase, which prefers to use full-length cDNAs as substrates for its terminal transferase. Moreover, SMART-Seq v4 is more efficient in template switching, which shortens the time of cDNA synthesis and cDNA library construction, and has a higher sensitivity for low input and higher reproducibility [28]. SMART-Seq v2 and v4 are widely utilized in cancer research, such as research on hepatocellular carcinoma (HCC) [29–31]. In addition, SMART-Seq does not require additional equipment, which means that it largely depends on experienced researchers.

A recent study compared sequencing data from CD45 cell samples generated by the 10X Genomics and SMART-Seq v2 platforms. In particular, SMART-Seq v2 was more sensitive and detected more genes within a single cell, especially low-abundance transcripts and alternatively spliced transcripts. The use of 10X Genomics increased dropout and showed increased noise in transcripts with low expression. However, 10X Genomics detected more genes due to its superior coverage of abundant cells, which thus enabled the identification of rare cell types. Further analysis of the sequencing data revealed that the two platforms detected distinct differentially expressed genes (DEGs) between cell clusters, indicating the potential to combine complementary results to interrogate heterogeneity [32]. Another study combining 10X Genomics and SMART-Seq v2 revealed the immune cell landscape and analyzed dynamic migration and status switch features in HCC [33].

Spatial transcriptomes and temporal lineage tracing facilitate multifaceted interrogation of the local environment and dynamic interactions within a single cell. Temporal and spatial variability influence tumor heterogeneity and stress reactions and are thus indispensable for cancer diagnosis, subtyping, classification and treatment in cancer research [34–36]. The sequential attachment of spatial barcodes makes it possible to encode and obtain location information on single cells, which can effectively provide information for use in research and diagnosis. For example, a recent study deciphered a detailed spatial map of single-cell phenotypes and cellular communities, which demonstrated phenotypic heterogeneity in the breast TME [37]. Furthermore, single-molecule RNA fluorescence in situ hybridization (smFISH) provides precise spatial information in pancreatic cancer and breast cancer [38, 39].

Cancer progression is a dynamic process that involves several different steps from oncogenesis and metastasis to the development of treatment resistance. Defining the temporal and molecular nature of each step in this process is critical to understanding cancer biology and designing effective treatment strategies. The newly developed technique lineage tracing by nuclease-activated editing of ubiquitous sequences (LINNAEUS) was used to reconstruct lineage trees in zebrafish with great success [40]. Single-cell analysis based on CRISPR-Cas9 technology was used to draw a detailed malignant cell spectrum in a KRAS mutant mouse model. This technique overcomes the difficulty of low mutation sensitivity, as well as the inability to decipher the changing details of tumor subtypes, which contributes to tracking the spread pattern and key genes in lung cancer. These novel findings indicated the potential for developing targeted therapy and improving the clinical management of lung cancer in patients with KRAS mutations [41]. Another study in which scRNA-seq and high-confidence clonal tracing were combined demonstrated the first detailed characterization of leukemic stem cells, shedding new light on the understating of leukemia oncogenesis and treatment [42].

Single-nucleus RNA sequencing (snRNA-seq) dates back to 2013, when Grindberg found that it was difficult to dissociate integrated cells from brain tissue [43]. Div-seq and DroNc-seq have been used to resolve the difficulty in identifying rare neural cells and obtaining integrated cell types [44, 45]. At the same time, researchers have conducted snRNA-seq analysis of brain tissues in autopsies to expand the cell landscape retrieved from scRNA-seq data, demonstrating that frozen samples can be analyzed by snRNA-seq [7]. Currently, snRNA-seq has been widely utilized for different tissues and cell types, such as the kidney, heart, lung, pancreas, and, especially, brain tissue [46–50]. Compared with scRNA-seq, scRNA-seq has several advantages. First, snRNA-seq can be utilized to analyze valuable frozen samples, as the nuclear membrane may remain integrated, unlike the cell membrane in frozen tissues [51]. Second, snRNA-seq does not introduce artifactual transcriptional stress responses or transcriptional bias, which may be induced by single-cell isolation in scRNA-seq, thus reflecting the real transcriptional status. Third, snRNA-seq can avoid the loss of specific cell types due to different vulnerabilities to proteases. Fourth, the preparation of a single nucleus is simpler than that of a single-cell suspension, which can minimize the generation of pseudocell populations induced by enzymatic hydrolysis and mechanical pressure.

SnRNA-seq has been widely utilized to analyze brain tumors because it is difficult to obtain fresh brain tissue for research, and the majority of samples are frozen. In 2020, researchers from MIT conducted scRNA-seq and snRNA-seq analyses of fresh or frozen tumor samples and evaluated the sequencing results with respect to cell and nuclear quality, cellular composition and other indicators. The comparison indicated that the two sequencing technologies detected similar cell types with distinct cellular proportions among different tissues. For example, the proportion of immune cells was higher, and the parenchymal cells (e.g., neural crest and neuroendocrine cells) were significantly reduced in neuroblastoma upon analysis by scRNA-seq. However, the proportion of substantial cells (especially malignant cells) was higher, while distinct immune cells were significantly reduced or even absent in the snRNA-seq data. Most importantly, significantly fewer neurons were identified by scRNA-seq than by snRNA-seq, which demonstrates the great potential of snRNA-seq in deciphering the landscape of brain tumors [52]. SnRNA-seq can obtain intron-region and intergene-region sequencing data, enabling cell type identification at a higher resolution and providing relatively richer gene information. However, the amount of RNA in a single nucleus is still significantly lower than that in whole cells, which suggests that snRNA-seq may not be appropriate for immune cell studies.

### Genomics

Single-cell DNA sequencing has not yet made the transition to high-dimensional analysis due to its high cost. Therefore, the more economic method is to apply bulk sequencing first, followed by targeted single-cell DNA sequencing of the mutations or variations of interest. For example, TARGET-seq, which integrates genomic DNA and coding DNA genotyping, achieves good coverage across key mutation hotspots and enables the highly sensitive analysis of mutations within single cells. However, TARGET-seq relies on an analysis of known mutations and does not support the identification of new mutations [53]. Additionally, the computational method Cardelino integrates clone tree information from bulk exome sequencing and infrequent variant alleles from scRNA-seq, delineates phenotypic variations between clones and has revealed DEGs between cancerous and healthy skin tissues involved in the cell cycle and proliferation pathways in [54].

### Epigenomics

Epigenetic dynamics can also be detected by single-cell sequencing technologies, such as chromatin immunoprecipitation sequencing (ChIP-seq) and assays for transposase-accessible chromatin using sequencing (ATAC-seq) [55]. In applying ATAC-seq to T cell receptor (TCR) research, transcript-indexed ATAC-seq (T-ATAC-seq) was reported to delineate the TCR specificity and epigenomic state of single T cells in cutaneous T cell lymphoma [56]. Furthermore, researchers introduced single-cell Cleavage Under Targets and Tagmentation (CUT&Tag) technology to probe the histone modification landscape in sequences including promoters, enhancers and gene bodies, as well as their dynamic regulatory interactions and single-cell chromatin occupancy, with high sensitivity and throughput within single cells [57].

To understand genome organization, researchers have used in situ genome sequencing (IGS) as a method for simultaneous in situ sequencing and imaging of genomes within intact single cells. IGS, which includes in situ genomics DNA library construction, in situ sequencing, amplicon dissociation, PCR and ex situ sequencing of amplicons and spatially localized sequences, reveals the precise localization of specific DNA sequences. IGS clearly provides a valuable opportunity for addressing intensive biological questions, such as the relationships between genome architecture and diseases [58]. Moreover, another study combined high-resolution multiple annealing and looping-based amplification cycles for digital transcriptomics (MALBAC-DT) and diploid chromatin conformation capture (Dip-C) to delineate transcriptomics dynamics and the three-dimensional genome architecture in single brain cells. This novel method could specifically decipher the roles of transcriptomics and genome architecture, as well as the interactions between function, anatomy, transcription and cell types in tumorigenesis and progression [59].

### Proteomics

Proteins represent the main functional machinery of cells, so deciphering the expressed proteome at the single-cell level has attracted great interest. Mass spectrometry is the basis for proteome detection and quantification; however, it is suitable for only the most abundant of proteins. Researchers have improved the procedures used for protein preparation and isolation, thus decreasing protein loss and facilitating more in-depth quantitative proteomics sequencing at a single-cell resolution. Mass cytometry by time of flight (CyTOF), which relies on metal isotope-labeled antibodies conjugated with specific signal molecules on the surface or inside cells for immunolabeling, allows the profiling of 100 distinct proteins in single cells. Imaging mass cytometry (IMC) was developed based on immunohistochemistry with metal-labeled antibodies, and CyTOF. IMC can simultaneously analyze up to 40 protein markers and their spatial architecture and interactions, information that would be lost by traditional tissue lysis to single cells [60]. Importantly, IMC can be performed with paraffin-embedded tissue sections, so it can be applied for retrospective analyses of patient cohorts whose outcomes are known, eventually benefitting personalized medicine. Another single-cell proteomics sequencing technology is liquid chromatography–mass spectrometry (LC–MS), a bioanalytical method for the quantitative analysis of proteins that is widely applied in biopharmaceutical drug development and drug toxicology studies. Recently, single-cell proteomics by mass spectrometry (SCoPE-MS) was introduced to quantify multiplexed single-cell proteomes [61]. As expected, single-cell proteomics sequencing will change the landscape of investigative pathology, particularly when used in coordination with multiomic platforms, such as transcriptomic and proteomic data, at a single-cell resolution.

### Multiomics

Progress in sequencing has enabled the integration of several sequencing technologies to delineate the TME and interactome within a single cell. First, simultaneous quantification of the DNA–protein interactome and transcriptome profile in a single cell would help researchers understand the transcriptional changes that occur when DNA binds a protein of interest, and such quantification has been realized by scDam&T-seq [62]. Second, concurrent sequencing of the transcriptome and targeted genomic regions (e.g., CORTAD-seq, G&T-Seq, and DR-Seq) within the same single cell can provide good coverage of the targeted genomic loci crucial for SNVs, deletion mutations and CNVs, which are responsible for resistance to targeted therapy in lung cancer [63]. The DNA methylome and mRNA transcriptome were simultaneously analyzed using scMT-Seq and scM&T-seq. Clone alignment statistically integrates independent single-cell RNA and DNA sequencing data [64]. Third, a recently reported integrative pipeline including 3D imaging based on the rapid clearing agent FUnGI was applied in breast tumors and revealed a significant reduction in tumor clones during oncogenesis, with the luminal progenitor found to be a key cell of origin [65]. LSR-3D imaging, together with multicolor lineage tracing and molecular analysis, provides essential visual and spatial information related to the tumor to study biological processes, highlighting the inherent plasticity of tumors. Fourth, several technologies, such as CITE-seq, REAP-seq and IN-seq, contribute to delineating the transcriptome and proteome at the same time [66].

## Analysis of single-cell sequencing data

Single-cell sequencing is widely used to detect DEGs and can detect key signature genes during tumor progression. Several bioinformatics tools are available to mine scRNA-seq data and provide valuable insights. For example, mixed isolated cells could be hierarchically clustered into various subsets based on DEGs along the pseudotime or CNVs, which can be used to construct clonality trees, as observed in medulloblastoma [67]. Second, SCENIC analysis based on gene expression levels could reveal the dynamic interactions between genes within single cells, construct a gene regulatory network and reveal the regulatory changes during phenotype switches. Third, analysis of enriched gene ontology terms and pathway analysis based on Kyoto Encyclopedia of Genes and Genomes could delineate the expression of genes involved in specific signaling pathways associated with tumorigenesis, the cell cycle, epithelial-to-mesenchymal transition (EMT) and immune responses among various subclusters. Notably, when expression information is combined with certain cell types, we can decipher the functions of the cell types during processes in tumor development, such as angiogenesis and tissue remodeling [68].

## Applications of single-cell sequencing in cancer biology

### The cancer cell atlas and malignant cell heterogeneity

In addition to the Human Cell Atlas, single-cell sequencing technology provides an unprecedented opportunity to decipher the functional states of single cancer cells. The accumulation of increasingly abundant single-cell sequencing data enabled establishment of The Cancer Cell Atlas, which covers a spectrum of cancers (Table 2). For example, Microwell-Seq contributed to cellular hierarchy construction and clonal heterogeneity deciphering in normal bone marrow and acute myeloid leukemia [69]. By integrating clinical pathological information and single-cell sequencing data, novel diagnostic and prognostic biomarkers and potential therapeutically relevant cell types or states could be deciphered [70]. In conclusion, The Cancer Cell Atlas can provide reference information for neoadjuvant therapy, especially for those for which baseline guidance is lacking [71].

**Table 2:** Establishment of the Cancer Cell Atlas by single-cell sequencing technologies

The atlas		Methodology	Key findings	References
Cancer specific	Spatial atlas of LUAD evolution	Single-cell RNA sequencing	Deciphered the geospatial evolution of cellular lineages, states and transcriptional features from normal tissue to LUAD. They also found that CD24 can mediate protumor phenotypes	[201]
	Ecosystem atlas in breast cancer	Single-cell RNA sequencing	Constructed the transcriptional atlas of the evolution trajectory from normal breast and preneoplastic BRCA1( ±) tissue to various subtypes of breast cancer, highlighting the significant heterogeneity in microenvironment	[202, 203]
	Infiltrated B cells in TNBC	Single-cell RNA sequencing and antigen receptor profiling	The presence of infiltrated B lymphocytes indicated the local differentiation within breast tumors and revealed the positive correlation between B cells and survival via potential immunosurveillance	[204]
	T cell atlas in gliomas	Single-cell RNA sequencing	Provided the landscape of tumor-infiltrating T cells of IDH wild-type and mutation glioma and identified CD161 as an immunotherapy target	[205]
	Immune cell atlas in PDAC	Single-cell RNA sequencing	Established the immune cell atlas in PDAC, which acts as a reference to evaluate the immune landscape and potential effect of immunotherapy	[71]
	Immune cell atlas in ESCC	Single-cell RNA sequencing and TCR sequencing	Demonstrated the dynamics of various immune cells along tumor progression and indicated several immunosuppressive mechanisms	[206]
	Cellular hierarchy atlas in AML	Microwell-Seq and SMRT-seq	Revealed the AML landscape and proposed a ‘cancer attractor’ phenotype, which may help define the AML progenitor cell associated with prognosis	[69]
Pancancer atlas	CancerSEA	Single-cell RNA sequencing	Provided a user-friendly database of 14 functional states of tumor cells (including stemness, invasion and EMT). It also provided the functional states associated PCG/lncRNA repertoires among cancers	[207]
	CD8 + T cell atlas	Transposase-accessible chromatin sequencing, RNA sequencing	Defined the differentiation trajectory of CD8 + T cells toward dysfunction and revealed the underlying transcriptional drivers across various tumors, including melanoma and HCC	[208]
	TIM atlas	Single-cell RNA sequencing	Revealed the similarity and distinction of TIMs, including mast cells, DCs and TAMs, across 15 tumors and revealed the association with somatic mutations and gene expression	[209]
	HLA atlas	Immunoaffinity purification and liquid chromatography mass spectrometry	Delineated the HLA-I and HLA-II immunopeptidomes from tumor and benign human tissue samples, enabling the balanced comparison of HLA ligand levels and thus facilitating immunotherapy	[210]
	Fibroblast atlas	Single-cell RNA sequencing	Demonstrated that fibroblast transcriptional states are conservative across species and in different diseases	[211]

LUAD: lung adenocarcinoma; IDH: isocitrate dehydrogenase; TIM: tumor-infiltrating myeloid cells; TAM: tumor-infiltrating macrophages; HLA: human leucocyte antigen; PDAC: pancreatic ductal adenocarcinoma; TNBC: triple-negative breast cancer; ESCC: esophageal squamous cell carcinoma; AML: acute myeloid leukemia; SMRT-seq: single-cell single-molecule real-time sequencing

The TME, which comprises cellular and noncellular components, plays crucial roles in tumorigenesis, progression, invasion, metastasis and drug resistance. These components orchestrate a tumor-promoting and tumor-inhibiting microenvironment that modulates tumor growth and influences tumor evolution, in which heterogeneity is also involved. The molecular basis for heterogeneity manifests as distinct malignant subpopulations based on structural variations, chromosomal rearrangement events, epigenetic modifications and gene expression signatures [72–77]. Moreover, nongenetic intratumor heterogeneity is a major predictor of phenotypic heterogeneity and evolutionary dynamics, rather than genomic features alone, in lung cancer [78]. However, heterogeneity constitutes the main obstacle to developing effective therapeutics; thus, deciphering the tumor tissue heterogeneity will substantially contribute to improving our understanding of the underlying mechanisms and developing precise therapies in clinical trials. Interestingly, recent findings showed that heterogeneity is stable over time across replicates of the same culture, strongly suggesting a regulated rather than a stochastic process, as described previously [79].

**Fig. 1:**
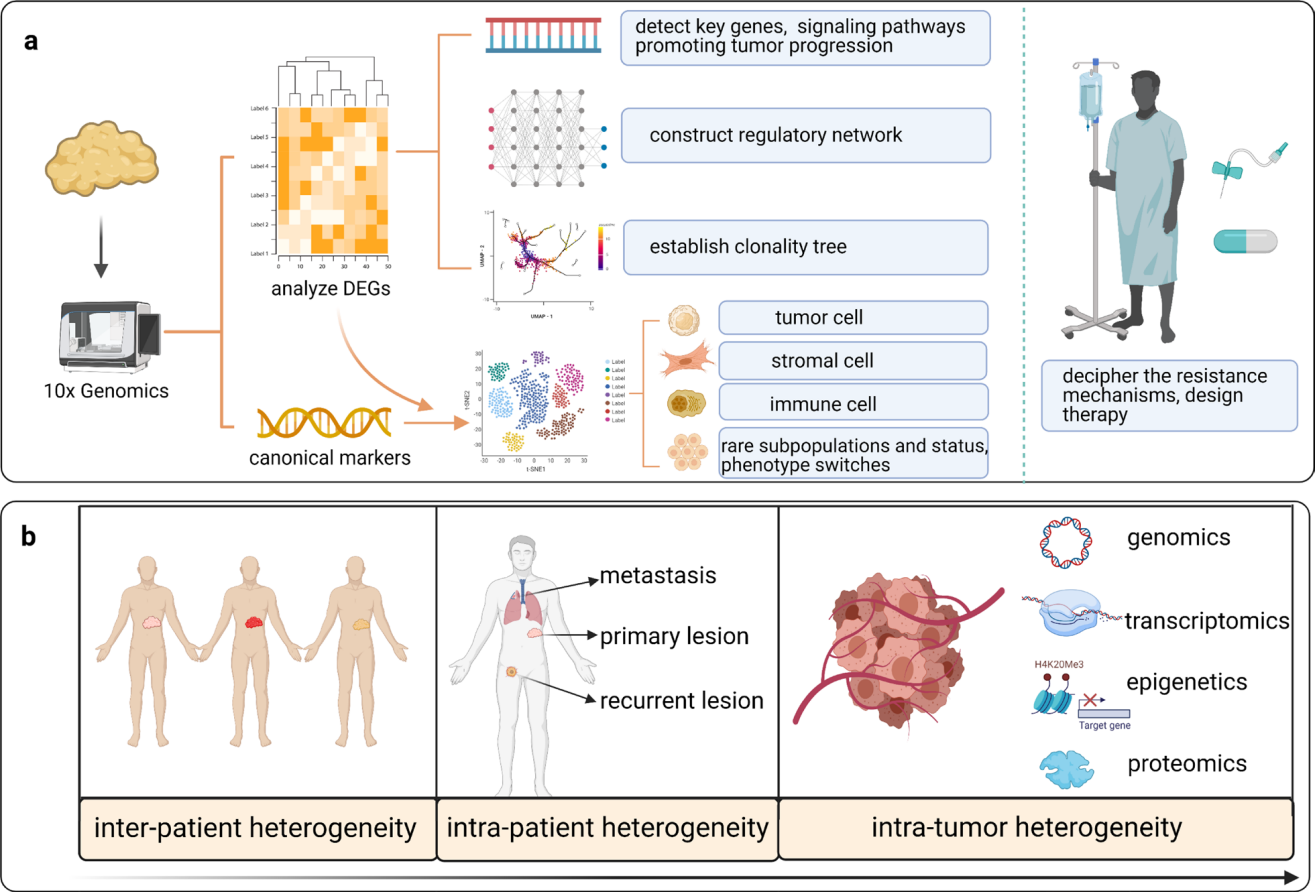
Application of single-cell sequencing in delineating tumor heterogeneity and designing novel targeted therapies for patients with various tumors. **a** Single-cell sequencing can be used to analyze differentially expressed genes (DEGs), thereby detecting key genes and signaling pathways that are altered during tumor progression and constructing a regulatory network and clonality trees within tumor lesions. When DEGs are combined with canonical markers, the cells are clustered, which enables the identification of rare subpopulations, cell states and phenotype switches during tumor progression. Interrogation of the tumor microenvironment (TME) and heterogeneity enables the disclosure of therapeutic resistance mechanisms and the design of novel therapies. **b** Single-cell sequencing explores tumor heterogeneity at distinct levels, including the population, individual cell, tissue and molecular levels

Compared to traditional bulk RNA sequencing, which reflects the average profiles of gene mutation and expression, scRNA-seq examines the multiomics features of individual cells, thus mapping the TME among various tumors, such as breast cancer [80]. These scRNA-seq data provide supporting evidence for accurate molecular subtyping and precise treatment (Fig. 1).

#### Identification of dynamic gene expression profiles during tumor progression

Single-cell sequencing can delineate changes in gene expression during tumor progression. Integrative single-cell sequencing of adjacent normal tissues and adenomas at different stages in patients comprehensively showed genomic alterations, clonal architecture and metabolic dynamics during tumorigenesis, providing insights into the inhibition of tumor progression [81]. For example, an evolutionary trajectory including the carcinogenesis of basal cells and activation of Wnt, followed by differentiation into luminal-like cells, was revealed in salivary gland squamous cell carcinoma [82]. Another example is the transformation of familial adenomatous polyposis to adenocarcinoma. Chen et al. discovered an intermediate epithelial–mesenchymal status, indicating that malignant cells retain epithelial characteristics while undergoing rapid migration in breast cancer [83]. A study of oncogene-induced senescence suggested a new mechanism of the tumor suppressor response to oncogene activation [84]. Interestingly, chromosome remodeling, including interwoven chromosome breakage–fusion–bridge cycles, micronucleation events and chromothripsis episodes, might also drive tumor evolution and cancer-associated thrombosis [85, 86].

### Identification of novel subpopulations, cell states and phenotype switches

Individual tumor cells tend to primarily cluster according to their patient origin, which is not surprising because each tumor has a specific evolutionary trajectory with interpatient heterogeneity. Meanwhile, scRNA-seq can cluster cells based on cell type with t-distributed stochastic neighbor embedding (t-SNE), which is an algorithm designed to deconvolve and visualize high-dimensional scRNA-seq data in an unsupervised manner. Principal component analysis (PCA) and canonical correlation analysis order the cells in a gradient to interrogate the features of the clusters. Generally, the cell clusters compromise malignant tumor cells, stromal cells (epithelial cells, endothelial cells, and cancer-associated fibroblasts (CAFs) and immune cells. Further subpopulation clustering contributes to defining rare subpopulations or a spectrum of cellular states of malignant cells. By combining an analysis of signaling pathways, we were able to identify the most malignant subtype associated with a poor prognosis, which may provide insights into targeted therapy for this subtype. Recently, scRNA-seq research identified melanoma cells in an intermediate state and a malignant cell subset with unique characteristics among pancreatic, ovarian and gastric cancer [79, 87–90]. Key genes identified during cellular transitions may be involved in transcriptional or posttranscriptional regulation and have the potential to serve as therapeutic targets. Among various malignant tumor cells, an analysis of the mean-squared displacement, velocity and maximal distance traveled contributes to the prediction of the migratory capacity of certain subpopulations, thus providing insights into the mechanisms of tumor metastasis. Unsupervised analyses of the transcriptional trajectory of malignant cells from Monocle2 may be useful for analyzing the dynamics of tumors. For example, malignant osteoclasts are split into progenitor cells, immature cells and mature cells in osteosarcoma [91].

The TME determines phenotypic features, thus enabling the best adaptation to the external environment. For example, malignant cells near the tumor tissue present characteristics of metastasis and invasion, while the malignant cells within the tumor maximize their proliferative ability by activating metabolism. Similar to next-generation sequencing, scRNA-seq can reveal phenotypic dynamics at different stages of tumor progression. Comparison of sequencing data from primary tumor lesions at different clinical stages, recurrent lesions, distant metastatic lesions and corresponding healthy tissue contributes to our understanding of the phenotypic changes that occur during tumor progression and metastasis, such as those in glioma, sarcoma, colorectal cancer and bladder cancer [92–94]. Phenotypic heterogeneity might be driven by transcriptomic heterogeneity through its effects on cancer-associated pathways. For example, a recent study revealed that malignant osteoblastic cells, which are characterized by high mesenchymal marker and transcription factor expression, may originate from any type of cell along the osteogenic differentiation pathway from mesenchymal stem cells in osteosarcoma [91]. MUC6 + TFF2 + spasmolytic polypeptide-expressing metaplasia, which may originate from gastric chief cells, is a state of precancerous lesions in gastric cancer [89]. In addition, oxidative phosphorylation plays a key role in breast cancer metastasis based on a comparison of primary tumors and micrometastatic tissue [95]. More key findings on the cell map of malignant cells are listed in Table 3.

**Table 3:** Key findings related to malignant cell heterogeneity among tumors obtained using single-cell sequencing

Tumor	Technology	Platform	Key findings	References
PDAC	scRNA-seq, trajectory analysis	10X Genomics	Identified 11 subpopulations of malignant tumor cells, including a subset of malignant ductal cells with unique proliferative features associated with the inactivation of tumor-infiltrating T cells	[90]
PDAC	scRNA-seq, trajectory analysis	10X Genomics	Identified 6 acinar metaplastic cell subpopulations during the progression from preinvasive stages to tumor formation in a mouse model	[212]
LUAD, LUSC	Bulk and scRNA-seq	10X Genomics	Revealed that nongenetic heterogeneity is a major predictor of phenotypic heterogeneity	[78]
LUAD	scRNA-seq	10X Genomics	Identified 19 tumor-specific markers as candidate markers for the detection of extraordinarily rare circulating tumor cells	[213]
lung cancer	Single-cell sequencing	CyTOF	Revealed that AXL inhibition suppresses SMAD4/TGFβ signaling and induces JAK1/STAT3 signaling pathways, and AXL functions via CD133-mediated cancer stemness and hybrid EMT features in patients with advanced-stage tumors	[214]
GGN-ADC, SADC	scRNA-seq, flow cytometry	10X Genomics	Revealed the downregulation of signaling pathways associated with angiogenesis and cell proliferation, low expression of collagens of fibroblasts and activated immune cells in GGN-ADC	[215]
melanoma	scRNA-seq, bulk RNA-seq, ATAC-seq	10X Genomics	Confirmed the intermediate state of melanoma cells, which is governed by SOX6, NFATC2, EGR3, ELF1 and ETV4 and regulated by a gene regulatory network	[79]
GA	scRNA-seq	10X Genomics	Identified 5 subtypes of malignant tumor cells, 3 of which corresponded with the histopathological features of Lauren’s subtypes, while 1 was associated with the GA-FG-CCP	[216]
EGC	scRNA-seq	10X Genomics	Revealed that the glandular mucous cells tended to acquire an intestinal-like stem cell phenotype during metaplasia; HSE6 may help identify early-stage tumors	[216]
AML	scRNA-seq, next-generation sequencing	10X Genomics	Identified 3 leukemia subpopulations arrested at different stages of myeloid differentiation: CD34*CD117^dim^ blasts (blocked in G0/G1 phase), CD34*CD117^bri^ blasts and partial maturation myeloid cells	[217]
MM	scRNA-seq	Fluidigm C1	Identified 4 groups of malignant cells; the L1 group from MGUS expressed the lowest levels of genes involved in oxidative phosphorylation, while the L4 group from MM displayed the highest expression	[218]
NEPC	scRNA-seq	10X Genomics	Revealed that focal neuroendocrine differentiation exclusively originates from luminal-like malignant cells and identified differentiation-association signature genes	[219]
medulloblastoma	scRNA-seq	10X Genomics	Identified OLIG2-expressing glial progenitors as transit-amplifying cells at tumor onset, and they are enriched in therapy-resistant and recurrent medulloblastoma	[220]
glioma	scRNA-seq	10X Genomics	Identified Zfp36l1 as a key gene for glioma growth, various transitional intermediate states and corresponding developmental lineages	[221]
glioblastoma	scRNA-seq	10X Genomics	Identified key genes involved in NSC transformation into tumor-promoting cells; NSCs may promote metastasis via extracellular vesicles	[222]
glioblastoma	scRNA-seq	10X Genomics	Identified RAD51AP1 as an independent prognostic factor and a potent mediator of EGFRvlll signaling	[223]
ESCC	scRNA-seq, bulk sequencing	SMART-Seq 2	Identified genes associated with radioresistance, including autophagy-related 9B, DNA damage-inducible transcript 4, myoglobin and plasminogen activator tissue type	[224]
HCC	scRNA-seq	10X Genomics	Identified the involvement of JUNB, a pro-oncogene, in the immune response and progression of HCC	[225]
HCC	Single-cell mass cytometry, RNA sequencing	Fluidigm	Revealed that the lncRNA HOXA-AS2 is associated with the regulation of cancer stemness during tumorigenesis and poor prognosis; identified an EPCAM + C-MYC + CK19 subpopulation in HOXA-AS2 high patients	[226]
breast cancer	scRNA-seq	Hydro-Seq	Revealed that CAFs increase cancer stemness and alter the epithelial/mesenchymal status during coculture with malignant cells	[111]
breast cancer	Single-cell whole-exome sequencing	Fluidigm C1	Revealed the evolutionary process of cells with SNVs in hit driver genes via CNVs acquired in chromosomal regions; identified the Plekha5 gene as a suppressor of tumor metastasis	[227]

PDAC: pancreatic ductal adenocarcinoma; LUAD: lung adenocarcinoma; LUSC: lung squamous cell carcinoma; GGN-ADC: ground glass nodule adenocarcinoma; SADC: solid adenocarcinoma; GA: gastric adenocarcinoma; GA-FG-CCP: fundic gland-type gastric adenocarcinoma; AML: acute myelogenous leukemia; NEPC: neuroendocrine prostate cancer; EGC: early gastric cancer; ESCC: esophageal squamous cell carcinoma; MM: multiple myeloma; HCC: hepatocellular carcinoma; ESCC: esophageal squamous cell carcinoma

#### CTCs and CSCs

CTCs, rare cancer cells that slough off from primary tumor tissues and circulate in the bloodstream, are thought to act as ‘seeds’ that initiate cancer progression and metastasis [96, 97]. Molecular analysis of CTCs at a single-cell resolution allows the generation of robust, rich genome and transcriptome datasets, thereby providing biomarkers to understand the underlying mechanisms of progression, prognosis, evaluation dynamics and therapeutic response [98–100]. CTCs and circulating tumor DNA have been approved by the US Food and Drug Administration as noninvasive liquid biopsies, and extracellular vesicles, circulating tumor RNA and tumor-educated platelets have been identified as novel constituents with promising potential in cancer management [99]. New platforms (e.g., Cheimera X-il20 and Hydro-Seq) were developed to overcome current limitations in the detection of CTCs, including cell loss, a high false-positive detection rate, CTC scarcity and substantial contamination with blood cells [101, 102].

**Fig. 2:**
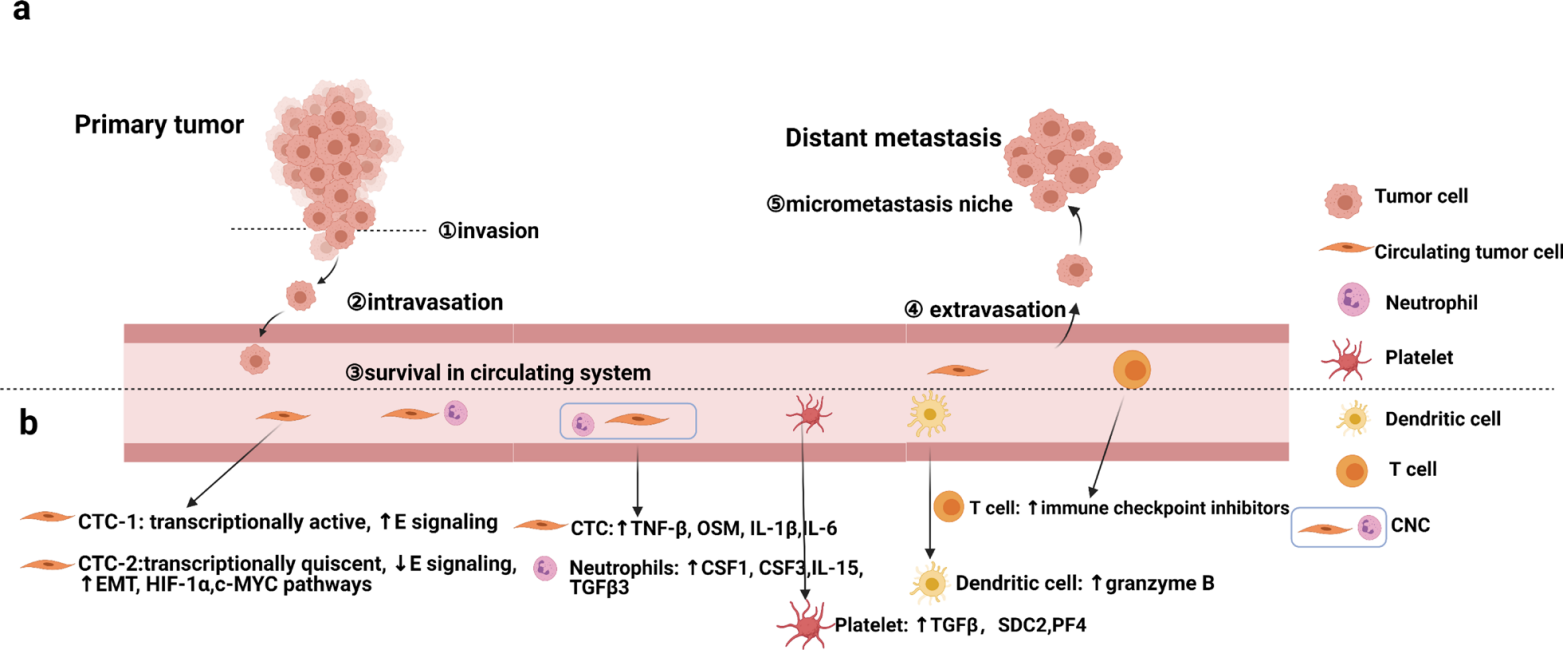
Schematic of tumor metastasis and interactions between circulating tumor cells (CTCs) and peripheral cells. **a** Tumor metastasis is a complex process that includes invasion of the primary tumor border, intravasation, survival in the circulatory system, extravasation and the formation of a micrometastatic niche in distant tissues. **b** However, CTCs in the circulatory system dynamically interact with peripheral cells, and CNCs are very important. E: estrogen; CTCs: circulating tumor cells; CNCs: circulating tumor cell and neutrophil niches

Based on accumulating evidence, CTCs travel in clusters rather than in isolation in the peripheral fluid [103]. The ability to provide insight into CTC subtypes and intravascular interactions with other blood cells may help elucidate the mechanisms of tumor progression and prognosis. In breast cancer, researchers identified 2 CTC subpopulations with differences in estrogen responsiveness and EMT, as well as the interactions between CTCs and peripheral monocytes [104]. Moreover, transforming growth factor β secreted by activated platelets is associated with an EMT phenotype in CTCs, contributing to escape from immune surveillance [97]. Furthermore, another scRNA-seq study revealed the presence of a neutrophil-CTC niche in peripheral blood. Neutrophils from this niche were found to express genes associated with the cell cycle and cytokines, such as TNF-β, OSM, IL-1β, IL-6 and Vcam1, at high levels, and these genes may be involved in niche formation [105]. Based on this finding, the CTC-neutrophil clusters in patients may be useful as a positive biomarker to predict disease outcomes, providing novel insights into targeted therapy for cancer (Fig. 2). Another study reported that the perivascular niche may act as a niche for various subtypes of brain tumor stem-like cells, while the microvascular tracks may serve as a path for tumor migration and therapeutic resistance [106].

CSCs exhibit self-renewal and multidirectional differentiation, and cancer stemness can be induced by chemotherapy rather than pure selection [107, 108]. Several signaling pathways (e.g., WNT, NOTCH, HIPPO, and RAS), hypoxia, and pathways related to DNA repair and the cell cycle were shown to be functionally associated with the induction of cancer stemness [109–111]. More recently, low proteasome gene expression was reported to promote the formation of CSCs via the NOTCH signaling pathway in malignancies [112]. Inhibition of the proteasome drove cancer cells to adopt a CSC state via the TGF-β signaling pathway [113, 114]. A subpopulation of CD24 + CD44 + cells was identified and found to be associated with cancer stemness in HCC [114]. Intriguingly, CSCs from distinct cancers exhibit variability with respect to surface markers, which increases the difficulty of developing CSC-targeted therapies [115].

#### Stromal heterogeneity

CAFs are major components of stromal cells in several tumors and influence the stroma by secreting excess extracellular matrix and interfering with collagen crosslinking, thus modulating tumor stiffness and facilitating cancer progression [116–121]. For example, cadherin 11 promotes extracellular matrix deposition to support the growth of pancreatic carcinoma and resistance to gemcitabine [122]. Moreover, CAF-derived factors may alter the immune microenvironment by inhibiting immune effector cell activity and recruiting immune-suppressive cells, allowing cancer cells to evade immune surveillance [123–128]. A recent sequencing analysis separated CAFs into 3 main subgroups: myofibroblastic CAFs, inflammatory CAFs and antigen-presenting CAFs. Specifically, a recent study revealed recurring, patient-dependent expression programs in stromal cells from primary hepatic duct carcinoma and hepatic metastatic tumors [129, 130].

### Immune cell heterogeneity and the immune microenvironment

The tumor immune microenvironment, which is an important component of the TME, exerts profound effects on the immunotherapeutic response and clinical outcome. The map of the immune atlas describes a growing portfolio of immune cells, including lymphocytes, monocytes, macrophages, natural killer cells and dendritic cells (DCs). Indeed, subpopulations comprising the immune microenvironment vary during tumor progression. For example, stromal and immune cells undergo dynamic ontological and functional changes that create a protumor and immunosuppressive microenvironment. Resident myeloid cells are gradually replaced with monocyte-derived macrophages and DCs upon T cell exhaustion in metastatic lung adenocarcinoma [68]. ScRNA-seq was applied to study promotion of the immunosuppressive microenvironment through changes such as the increased expression of protein disulfide isomerase family A member 3 (PDIA3) associated with PTEN loss and EGFR amplification in gliomas [131]. Additionally, the cellular composition is associated with prototypic genetic lesions, as observed for the abundance of FLT3-ITD and abundant progenitor-like cells in acute myelocytic leukemia [9]. Research on the mechanisms of tumor heterogeneity has the potential for use in stratified targeted therapy according to tumor category and stage. Moreover, we have summarized recent findings of the tumor immune microenvironment in Table 4.

**Table 4:** Key findings related to immune cells within various tumor lesions obtained using single-cell sequencing

Tumor	Technology	Platform	Key findings	References
osteosarcoma	scRNA-seq	10X Genomics	Identified proinflammatory FABP4 + macrophage infiltration in lung metastatic lesions and revealed that TIGIT blockade enhances the effects of the primary CD3 + T cells	[91]
LUAD, LUSC	Bulk and scRNA-seq	10X Genomics	Revealed that the neoantigen burden is negatively associated with immune cell infiltration and affects evolutionary dynamics	[78]
RCC	scRNA-seq	10X Genomics	T cell exhaustion is a key factor that represses immunity; PD-L1, LAG3, TIM-3 are potential targets	[56]
RCC	scRNA-seq, flow cytometry	10X Genomics	The baseline and dynamic renal cell carcinoma tumor burden influence the T cell repertoire; baseline TCR β-China is associated with the prognosis	[154]
CAC	scRNA-seq, flow cytometry	10X Genomics	Identified the transcriptomic signatures of CD4 + T cells between antitumor and anti-viral infection responses	[155]
cHL	scRNA-seq, imaging mass cytometry	CyTOF	Identified LAG3 + Tregs in MHC-II-negative classic HL	[134]
HCC	scRNA-seq	10X Genomics, CyTOF	Identified the trajectory and functional analysis of CD4/CD8 double-positive T cells enriched in L regions with synergetic expression of PD-1/HLA-DR/ICOS/CD45RO and certain transcription factors	[142]
HCC	scRNA-seq	10X Genomics	Identified the abnormal immune cells contents dynamics in relapse tumors	[137]
HCC	scRNA-seq, flow cytometry	10X Genomics	Identified the M2 macrophages that expressed CCL18 and CREM at high levels, as well as XCL1 + activated T cell subsets as a ‘pre-exhaustion’ status	[136]
HCC	scRNA-seq	10X Genomics, SMART-Seq 2	Identified the enrichment of MIAT in FOXP3 + CD4 + T cells and PDCD1 + /GZMK + CD8 + T cells, which modulates the expression of distinct genes (JAK2, SLC6A6, KCND1, MEIS3 or RIN1) to contribute to immune escape	[228]
CTCL	scRNA-seq	10X Genomics	Identified the heterogeneity of malignant T cells	[229]
CTCL	scRNA-seq, flow cytometry	10X Genomics	Identified the FOXP3 + T cells transiting to GATA3 + or IKZF2 + tumor cells during clonal evolution, which could be used to classify the tumor stage and predict early-stage progression	[230]
Osteosarcoma	scRNA-seq	10X Genomics	Revealed that TIGIT blockade enhances the cytotoxic effects of the primary CD3 + T cells on advanced osteosarcoma	[91]
Lung cancer	scRNA-seq, flow cytometry	10X Genomics	Compared the cancer cells and CD3 + T cells between GGN-ADC and SADC	[215]
Breast caner	scRNA-seq	10X Genomics	Identified the characteristics of myeloid-derived suppressor cells	[231]
PDAC	scRNA-seq	10X Genomics	Identified a subset of ductal cells with proliferative features associated with inactivating T cells	[49]
GBM	Single-cell and bulk RNA sequencing	10X Genomics	Revealed the molecular determinants, including TLE2 and IKZF2, required to improve the CAR antitumor efficacy; identified the genes sensitive to CART therapy	[232]
MM	scRNA-seq, flow cytometry	10X Genomics	Identified the myeloma cells equipped with an immune invasion ability due to the upregulation of inhibitory molecules for cytotoxic T cells and NK cells	[233]
CRC	scRNA-seq	Illumina Bio-Rad	Revealed the increased infiltration of granulocytes and the underlying mechanisms, which are associated with ferroptosis-mediated cell death and Wnt signaling pathway activation in colorectal cancer liver metastases	[234]
NSCLC	scRNA-seq, flow cytometry	10X Genomics	Identified 25 tumor-infiltrating myeloid cell states that are conserved in tumor lesions from patients	[10]
B cell malignancies	scRNA-seq, TCRB seq	10X Genomics	Disclosed the transcriptional programs identifying the clones after CAR-T cell infusions that mainly originated from infused clusters with high expression of cytotoxicity- and proliferation-related genes	[235]

RCC: renal cell carcinoma; HCC: hepatocellular carcinoma; CAC: colon adenocarcinoma; cHL: classic Hodgkin lymphoma; CTCL: cutaneous T cell lymphoma; NSCLC: non-small-cell lung cancer; PDAC: pancreatic ductal adenocarcinoma; CyTOF: cytometry by time-of-flight; MIAT: myocardial infarction-associated transcript; GBM: glioblastoma; CAR: chimeric antigen receptor; MM: multiple myeloma

#### T cells

Distinct types of T cells orchestrate antitumor T cell responses to fight malignancies in cooperation with other immune cells. Tumor-infiltrating lymphocytes (TILs) are a highly heterogeneous population in terms of cell type composition, molecular subtype, gene and protein expression and functional properties at different stages; thus, a systematic interrogation of TILs is key to the development of immunotherapies [132]. Importantly, scRNA-seq provides information about cellular distribution, clonal amplification, migration and evolution dynamics, as well as a map of the immune landscape through TCR analysis. According to recent studies, some TILs, acting as ‘bystanders,’ are not capable of recognizing antigen-bound major histocompatibility complex (MHC) molecules and then attacking tumor cells, suggesting that the TIL content is not related to the predicted responsiveness of immunotherapy among tumors. Aoki et al. identified a disease-defining T cell subpopulation with high expression of LAG3, which functions to mediate immunosuppression in classic Hodgkin lymphoma [133]. In tumor tissues, regulatory T cells (Tregs) undergo abnormal changes in expression, such as the change in LAG3 expression observed in classic Hodgkin lymphoma, upregulated LAYN expression in FOXP3 + Helios + Tregs, and changes in CCR8 and IL1R2 expression in HCC [134, 135]. In addition, the chronic remodeling of tumor antigens alters Treg cell frequencies and subsequently promotes the recurrence of HCC [136, 137].

T cells are the main components of the adaptive immune system responsible for antitumor immunity and responsiveness to immunotherapy. Single-cell analyses ignore the complex interactions between different cells, and by analyzing the correlations of ligand-receptor pair expression levels among various cell types, we can decipher the landscape of these cellular interactions [138, 139]. Heterodimeric TCRs that consist of two different subunits determine biological functions by interacting with various ligands, and the genetic recombination of subunits creates a diverse TCR repertoire [140]. Moreover, tumor neoantigens must be presented by MHCs and then recognized by TCRs on TILs. The combined use of scRNA-seq with TCR sequencing enables the identification of rare immune subpopulations and their potential functions and links T cell phenotypes with the specific TCR clonotypes of individual T cells. For example, the newly identified CD4 + CXCL13 + BHLHe40 + Th1-like CD4 + subset might explain the difference in responses to anti-PD1 therapy in patients with colorectal cancer presenting with MSI and MSS, while PD-1^high^ CD4 + CD8 + T cells are associated with prolonged survival in patients with HCC [141–143]. Integrated scRNA-seq and scTCR-seq analyses revealed the clonotypic expansion of effector-like T cells in tumors and tumor-adjacent tissues, even in peripheral blood. In addition, in immune circulation, intratumor T cells are replenished with fresh, nonexhausted cells from outside the tumor instead of cells within the tumors [144].

#### B cells

Malignant B cells exhibiting specific transcriptomic profiles are mainly present in lymphoma. The newly identified continuum of cyclic germinal center B cell transitional states reveals the heterogeneity in follicular lymphoma [145, 146]. However, B cell heterogeneity is somewhat determined by somatic mutations, indicating the induction of other mechanisms, including phenotypic diversity or epigenetic modifications [145]. Integrated single-cell sequencing and trajectory analyses also enabled researchers to decipher the evolutionary pathway of malignant B cell maturation, which is accompanied by the progressive loss of follicular and germinal center B cell gene expression programs (e.g., KMT2D, CREBBP and EZH2) [146]. Other researchers further explored the relationship between intratumor heterogeneity and treatment response using a combination of scRNA-seq with transcriptome-informed flow cytometry and identified subpopulation-specific drug sensitivity in nodal B cell lymphoma [147].

#### Monocytes

Myeloid cells, including tumor-associated macrophages (TAMs) and DCs, have been demonstrated to control tumor malignancy [148]. Myeloid cells exhibit changes in transcriptional pattern and infiltration extent and show great heterogeneity among various tumors.

TAMs display noticeable plasticity in their phenotypic and functional properties after their derivation from the mononuclear phagocyte system. To the best of our knowledge, TAMs undergo polarized activation upon distinct exposure. Some single-cell sequencing studies demonstrated that the classically activated (M1) macrophage signature and alternatively activated (M2) macrophage signature could coexist in single macrophages, thus identifying various subsets of myeloid cells [136, 149]. Apart from the traditional M1/M2 activation paradigm in macrophages, scRNA-seq has revealed the complexity of macrophage expression profiling. For example, scRNA-seq with CITE-seq revealed that TAMs derived from microglia (Mg-TAMs) or classical monocytes (Mo-TAMs) exhibit a spectrum of transcriptional activation states. However, they exhibited a convergent angiogenic and T cell-suppressive capacity ex vivo in glioblastoma [149].

Although DCs represent only a small proportion of leukocytes, they play an important role via their strong antigen-presentation capacity. ScRNA-seq analysis of the intratumoral heterogeneity of DC subsets identified transcriptomic signatures and the prognostic value of DCs [150]. For example, LAMP3 + DC clusters, which migrate from tumors to hepatic lymph nodes, appear to be the most active immune regulators due to their secretion of the most distinct immune-related ligands and modulation of the phenotypes of lymphocytes [33]. These findings indicate that targeting specific TAM and DC subsets may hold significant diagnostic, therapeutic and prognostic potential.

#### Immunotherapy

In recent years, the development of immunotherapy has significantly enhanced cancer therapies, and immune checkpoint inhibitors (ICIs) have had tremendous effects on some advanced tumors [151]. However, the clinical responses to immunotherapy vary among different patients and cancer types, as evidenced by the limited clinical success rate in patients with HCC [152]. Furthermore, ICIs alone were reported to display limited efficacy, ranging from 15 to 30%, except in melanoma [153]. ScRNA-seq and various multimodal techniques derived from scRNA-seq are advancing our understanding of human tumor heterogeneity and the mechanisms that drive responsiveness and resistance to immunotherapies, thereby providing insights into potential combinational therapies for different cancers [36].

Exhausted T cells are characterized by the high expression of inhibitory receptors, including PD-1, CTLA-4, TIM3 and LAG3, and hierarchical loss of effector functions, which is the target of ICI therapy [154]. Activated CD8 + T cells that express XCL1 at high levels are characterized as in a potential “pre-exhaustion” status and may function by recruiting cDC1 cells [136]. Moreover, the heterogeneity of CD4 + T cells might partially explain their distinct responses to tumor antigens and viral infection [155]. Researchers have identified other immune cells that participate in immunotherapy. For example, lenvatinib presents antitumor activity by reducing TAM infiltration and activating the interferon pathway in HCC [156]. Improved vascularity was shown to result in an improved pancreatic ductal adenocarcinoma (PDAC) prognosis, possibly due to the increased infiltration of immune cells and/or delivery of drugs [157]. These profound results have contributed to the screening of specific targets for immunotherapy. By performing single-cell sequencing in patients treated with ICIs, we were able to analyze the specific transcriptional activity of ICIs or select neoantigens transcribed at a high level, enabling the design of precise therapy. By analyzing the heterogeneity of immune-related genes using scRNA-seq, we can design combination therapies that target various tumor neoantigens and thus improve clinical efficacy.

A comprehensive understanding of the immune cell composition and states is crucial to delineating the responsiveness and resistance to immunotherapies and could help in the design of novel targeted immune-modulating therapies. Notably, the identification of transcriptome-wide signatures associated with therapeutic response or resistance improves our understanding of the mechanisms of immunotherapy. Extending the transcriptional immune atlas of normal tissue to the TME could provide intriguing insights into the heterogeneity and distinct immunotherapeutic responses of a spectrum of tumors. For patients who are insensitive to immunotherapy (such as CTLA4 and PD1/PD-L1), single-cell sequencing might be able to analyze specific tumor tissues and elucidate the underlying mechanisms of resistance. For example, a new subset of macrophages with high CD73 expression was shown to be associated with a reduction in immune cell infiltration. Therefore, targeted therapy could combine CD73 blockers with PD-1/CTLA-4, a potential strategy of clinical significance [158]. Another example is the construction of an immune checkpoint gene network in patients with follicular lymphoma who were insensitive to CTLA-4/PD-1 therapy, which improved our understanding of the complex mechanisms underlying immune invasion and provided new insights into therapy design [145].

According to Darwinian theory, resistant cells are thought to arise from selection pressure on heterogeneous malignant cells. However, recent studies have shown that simple selection does not adequately account for late relapse, and resistance patterns (e.g., transcriptomic reprogramming) may exist before treatment across a spectrum of tumors [159–163]. In a PD-1-resistant colorectal cancer patient with high microsatellite instability, biallelic loss of the beta2-microglobulin (β2M) gene was found to be associated with intrinsic resistance [164]. Moreover, a precise pattern, including decreased NF-κB-binding signatures, followed by a rapid reduction in the regulatory activity of transcription factors (e.g., EBF1, FOXM1 and IRF4), was revealed in chronic lymphocytic leukemia patients with ibrutinib resistance [165–167]. This approach disentangles the dispute over whether resistance is the result of selection or transcriptional adaption, providing valuable insight into the development of drug resistance and the application of stage-specific biomarkers. Nevertheless, epigenetic alterations, including those due to KDM5 histone demethylase activity, were identified as correlated with therapeutic resistance by Kunihiko [168].

ScRNA-seq has also been used to explore the novel combination of immunotherapy with chemical or radiation therapy to improve clinical effects among patients with cancer and explore the relationship of resistance between distinct therapies [169, 170]. For example, radioresistant cells from basal breast cancer with a high tumor mutation burden, high degree of mutational microsatellite instability and activated NRF2 pathways exhibit a higher rate of PD-L1 positivity [171]. In TME-modulation strategies, traditional delivery systems limit drug retention times, and nanoparticles with unique physical properties and elaborate designs efficiently penetrate the TME and specifically deliver drugs to targeted components of the TME among a spectrum of tumors. However, researchers’ interest in recent years has increasingly focused on the toxicity of monoclonal antibodies. A recent study revealed the genes (e.g., SPP1, HMOX1, TIMP1 and NAMPT) associated with trastuzumab-mediated cardiotoxicity in breast cancer [172]. These findings are useful for identifying potential prognostic biomarkers and expanding the immunotherapy scope based on patient subtype and dominant immune cell crosstalk, paving the way for the use of combination therapy in patients with cancer.

### Diagnosis

Cancers are traditionally diagnosed based on tissue origin and histologic features. Growing evidence has shown that molecular features influence the TME and thus alter clinical behaviors and responses to therapy. A better understanding of molecular features through identifying sensitive biomarkers, mutations or gene expression profiles will improve the diagnosis and targeted therapy of cancer in patients. For example, a malignant cell subpopulation dominating the metastatic stage was demonstrated to be a biomarker in lung cancer, while a tumor-associated microglia/macrophage-mediated EGFR/ERBB2 feedback-crosstalk signaling module was proven to outperform traditional gene biomarkers in glioma [68, 173]. Another potential diagnostic direction involves the molecular subtype among various tumors, such as bladder cancer and pancreatic cancer [174]. A recent study in bladder cancer correlated scRNA-seq data with previously known molecular subtype information (e.g., luminal-papillary, luminal-infiltrated, luminal, basal-squamous and neuronal) and revealed the correlation between molecular and clinical features, guiding molecular diagnosis and targeted therapy [175]. In addition, the detection of CTCs by single-cell sequencing technology contributes to early diagnosis and prognostic monitoring.

#### Treatment

Single-cell sequencing is widely utilized to measure the clinical effectiveness and safety of novel drugs in clinical trials. A search for ‘cancer’ and ‘single-cell sequencing’ on the http://clinicaltrails.gov Web site yielded 16 relevant clinical trial records (Table 5). Most of these records described phase 2 clinical trials. Single-cell sequencing can be used to explore dynamic regulatory networks and identify heterogeneous cellular behaviors in response to various chemical drugs or the irradiation of cell lines or tumor samples, such as esophageal squamous cell carcinoma samples [176]. The newly developed technique sci-Plex, which is based on Sci-Seq and nuclear hashing, has been used to decipher transcriptomic changes in millions of malignant cells toward distinct chemical drugs and the underlying mechanisms. Compared with traditional high-throughput chemical screens, sci-Plex can delineate more nuanced molecular mechanisms and reveal transcriptomic trajectories behind drug responses at a single-cell resolution with a lower cost [177].

**Table 5:** Clinical trials for cancer treatments associated with single-cell sequencing

NCT number	Phase	Tumor	Title	Status	Primary outcome measures	Single-cell sequencing-related outcome	Enrollment
NCT03117751	Phase 2|Phase 3	acute lymphoblastic leukemia and lymphoma	Total Therapy XVII for Newly Diagnosed Patients With Acute Lymphoblastic Leukemia and Lymphoma	Recruiting	Event-free survival (EFS) of patients with ALL	Single-cell sequencing to monitor somatic mutations in peripheral blood as patients undergo treatment	1000
NCT04352777	Phase 2	breast cancer	Impact of Endocrine Therapy and Abemaciclib on Host and Tumor Immune Cell Repertoire/Function in Advanced ER + /HER2- Breast Cancer	Recruiting	Changes in serum estrogen (E1 and E2) levels compared to changes in the tumor immune cell repertoire and function in response to endocrine therapy and CDK 4/6 inhibition	Changes in tumor immune cell populations will be assessed using scRNA-seq	30
NCT03984578	Phase 2	colorectal cancer	Window of Opportunity Study in Colorectal Cancer	Recruiting	Tumor immune gene expression signature|Pathological regression	Relative proportion/percentage of different immune cell states or immune cell types as inferred from single-cell profiling	50
NCT04460248	Phase 2	diffuse large B cell lymphoma	Zanubrutinib, Lenalidomide and Rituximab (ZR2) in Elderly Treatment-naïve Patients With Diffuse Large B-cell Lymphoma (DLBCL)	Recruiting	Complete response rate	scRNA-seq of tumor tissues	40
NCT03921021	Phase 2	esophagogastric adenocarcinoma	Phase 2 Study of Telomelysin (OBP-301) in Combination With Pembrolizumab in Esophagogastric Adenocarcinoma	Recruiting	Overall response rate, as assessed by radiographic imaging	Change from baseline in the tumor-immune microenvironment measured using scRNA-seq	41
NCT04367025	Phase 2	gastric cancer	Efficacy and Safety of Perioperative Chemotherapy Plus PD-1 Antibody in Gastric Cancer	Not yet recruiting	Major pathological response (MPR)	Differences in T cell gene expression were detected using scRNA-seq to screen people who were more sensitive to immunotherapy	70
NCT04656535	Early Phase 1	glioblastoma	AB154 Combined With AB122 for Recurrent Glioblastoma	Not yet recruiting	Incidence of treatment-emergent adverse events [safety and tolerability] associated with the combination AB122 and AB154 in patients with recurrent glioblastoma	scRNA-seq of tumor and blood after exposure to AB154 with and without AB122	46
NCT03655444	Phase 1|Phase 2	head and neck squamous cell carcinoma	Abemaciclib + Nivolumab in Patients With Recurrent/Metastatic Head and Neck Squamous Cell Carcinoma That Progressed or Recurred Within Six Months After Platinum-based Chemotherapy	Terminated	Phase I Only: Determine the recommended phase 2 Dose of abemaciclib combined with a fixed dose of nivolumab|Overall survival (OS) rate	scRNA-seq analysis of tumor tissue and blood obtained before and during treatment with abemaciclib and nivolumab	6
NCT04588038	Phase 1	head and neck squamous cell carcinoma	NT-I7 for the Treatment of Recurrent Squamous Cell Carcinoma of Head and Neck Undergoing Surgery	Not yet recruiting	Proportion of treatment-related adverse events	Gene expression profiling using scRNA-seq	10
NCT03869034	Phase 2	hepatocellular carcinoma	TAI Combined With PD-1 Inhibitor in Locally Advanced, Potentially Resectable HCC	Active, not recruiting	Progression-free survival (PFS) assessed using RECIST 1.1 criteria	Biomarkers of treatment response by single-cell RNA sequencing	40
NCT03407170	Phase 2	melanoma	Immunologic Determinants of Response to Pembrolizumab (MK-3475) in Advanced Melanoma (MK-3475–161/KEYNOTE-161)	Terminated	Mean fraction of cytotoxic T lymphocytes (FCT) in participants who achieved a response compared with participants who experienced progression	Neoepitope sequencing will be generated based on scRNA-seq	1
NCT03534635	Phase 2	melanoma	Analysis of the Modulation of the Tumor Microenvironment by MK-3475 (Pembrolizumab) Using a Systems Biology Approach (PEMSYS)	Recruiting	Identification of biomarkers using genomics and proteomics tools (scRNA-seq, exome sequencing, and single-cell profiling), multiplexed immunohistochemistry and bioinformatics	Identification of biomarkers using scRNA-seq	30
NCT03743766	Phase 2	melanoma	Nivolumab, BMS-936558 in Combination With Relatlimab, BMS-986016 in Patients With Metastatic Melanoma Naïve to Prior Immunotherapy in the Metastatic Setting	Recruiting	Change in LAG3 expression	scRNA-seq	42
NCT04217317	Phase 2	non-Hodgkin lymphoma	CPI-613 in Combination With Bendamustine in Patients With Relapsed/Refractory T-Cell Non-Hodgkin Lymphoma	Recruiting	Number of participants who successfully complete the therapy regimen	Single-cell sequencing	12
NCT04697940	Phase 1|Phase 2	non-Hodgkin lymphoma	Decitabine-primed Tandem CD19/CD20 CAR T Cells Treatment in r/r B-NHL	Recruiting	Safety in phase 1	Analysis of CAR T cell populations from patients using single-cell sequencing to determine distinct subtypes and clonal expansion of infiltrating lymphocytes	30
NCT04495894	Early Phase 1	non-small-cell lung cancer and renal cell carcinoma	Pre-Incisional Ketorolac for Patients Undergoing Surgery for Non-Small-Cell Lung Cancer and Renal Cell Carcinoma	Recruiting	Incidence of blood transfusions among the ketorolac group	scRNA-seq	76

Angiogenesis-targeted therapy has been demonstrated to play a key role in modulating tumor heterogeneity and tumor liver metastasis, and the underlying metastatic patterns were recently revealed by scRNA-seq [178–180]. The combination of VEGF blockade and tyrosine kinase inhibitors has shown promising clinical effects in advanced tumors, such as HCC and pancreatic neuroendocrine tumors [181, 182]. Complex growth patterns may explain why the antiangiogenic therapy efficacy varies among patients and thereby may function as a stratification method to recognize populations who might experience a benefit.

## Conclusions

Single-cell sequencing has advanced at a very rapid speed, and emerging and powerful technologies will undoubtedly promote new levels of precision and accuracy in molecular cancer research, such as the tumor microenvironment and heterogeneity. Therefore, it significantly improved our understanding of cancer diagnostic stratification, biomarkers, precise treatment and prognosis prediction [183–188].

However, the limitations of single-cell sequencing should be recognized. First, scRNA-seq intrinsically shows that not all eukaryotic cells undergo transcription at a consistent basal rate. Transcription occurs in pulses; therefore, instant sequencing is unable to completely decipher the transcription map [189, 190]. Additionally, most sequencing methods are designed for 3′ or 5′ reads and are not sensitive to transcripts at low abundance. Second, scRNA-seq alone cannot correlate genotype and phenotype, suggesting the need for high-throughput, low-cost multiomic technologies to delineate the overall tumor tissue landscape. Third, batch effects derived from the use of different platforms and processing procedures and analysis on different days might exist. Such batch effects are extremely clearly when different data from different sequencing studies are analyzed. Finally, this type of sequencing analysis is challenging to implement in larger cohorts; thus, existing findings should be applied with caution and confirmed in clinical trials. Moreover, the individual differences among patients and different platforms applied in distinct trials limit the reliability of the results described above.

Multiomics integrates high-throughput biomolecular data related to multiple factors, including the genome, transcriptome, proteome, interactome, epigenome and metabolism, among which the transcriptome is central. The multiomics data promote the interrogation of complex interactions and connections in tumors at different states and with different phenotypes and also provides additional nuanced data on the dynamic progression of tumor initiation, progression, growth, immune evasion, metastasis, relapse and therapy resistance. The integration of multiomics methods improves the technical per-cell reliability and sensitivity, which results in superior accuracy, robustness, compatibility and expandability and even improves the preamplification procedure. Overall, these data contribute to establishing a complex computational framework, which enables the de novo discovery of both continuous and discrete expression patterns from single-cell sequencing data.

In conclusion, the integration of sequencing techniques can generate more complex, high-throughput information, including genomic, epigenomic, transcriptomic, proteomic, spatial and temporal data, necessitating the development of powerful and precise models or algorithms to provide new strategies for diagnosis, classification, targeted treatment and prognosis prediction. Notably, the accuracy and sensitivity of emerging technologies and computational analyses must be improved, and the costs should become more affordable in the near future.

## Data Availability

Not applicable; all information in this review can be found in the reference list.

## Abbreviations

SCS: Single-cell sequencing
snRNA-seq: Single-nucleus RNA sequencing
Sci-Seq: Single-cell genomes with combinatorial indexing
RT: Reverse transcription
SPLiT-seq: Split-pool ligation-based transcriptome sequencing
FACS: Fluorescence-activated cell sorting
MARS-seq: Massively parallel single-cell RNA sequencing
inDrop: Indexing drop RNA sequencing
ScATAC-seq: Single-cell transposase-accessible chromatin using sequencing
CoBATCH: Combinatorial barcoding and targeted chromatin release
PAT: Tn5 transposase to protein A
CITE-seq: Cellular indexing of transcriptomes and epitopes by sequencing
REAP-seq: RNA expression and protein sequencing assay
INs-seq: Intracellular staining and sequencing
SMART: Switching Mechanism at 5′ End of RNA Template
TSO: Template switch oligo
IGS: In situ sequencing
CyTOF: Mass cytometry by time of flight
IMC: Imaging mass cytometry
LC–MS: Liquid chromatography–mass spectrometry
SCoPE-MS: Single-cell proteomics by mass spectrometry
t-SNE: T-distributed Stochastic Neighbor Embedding
PCA: Principal component analysis
CCA: Canonical correlation analysis
CNV: Copy number variation
DEG: Differently expressed gene
CAF: Cancer-associated fibroblast
GA: Gastric adenocarcinoma
LCM: Laser-capture microdissection
LINNAEUS: Lineage tracing by nuclease-activated editing of ubiquitous sequences
TCR: T cell receptor
ScRNA-seq: Single-cell RNA sequencing
SNV: Single-nucleotide variation
CNV: Copy number variation
Sv: Structural variation
CTC: Circulating tumor cell
CSC: Cancer stem cell
TME: Tumor microenvironment
TGF-β1: Transforming growth factor β1
PDIA3: Protein disulfide isomerase family A member 3
PDAC: Pancreatic ductal adenocarcinoma
HCC: Hepatocellular carcinoma
ESCC: Esophageal squamous cell carcinoma
CAF: Cancer-associated fibroblast
TIL: Tumor-infiltrating lymphocyte
MSI: Microsatellite instability
MSS: Microsatellite stability
PD-1: Programmed death 1
CTLA-4: Cytotoxic T-lymphocyte-associated protein 4
DC: Dendritic cell
cDC: Conventional type 1 dendritic cell
Treg: Regulatory T cell
MIAT: Myocardial infarction associated transcript
CRISP-seq: ScRNA-seq on cluster regulatory interspaced short palindromic repeats–pooled screens
TAM: Tumor-associated macrophages
M1: Classically activated macrophages
M2: Alternatively activated macrophages
EMT: Epithelial-to-mesenchymal transition
MET: Mesenchymal-to-epithelial transition
ICI: Immune checkpoint inhibitor
SmFISH: Single-molecule RNA fluorescence in situ hybridization
G&T-Seq: Genome and transcriptome sequencing
DR-Seq: DNA-mRNA sequencing
T-ATAC-seq: Transcript-indexed ATAC-seq
CORTAD-seq: Concurrent sequencing of the transcriptome and targeted genomic regions
scDam&T-seq: Single-cell DNA adenine methyltransferase identification with messenger RNA sequencing
